# Heart beat but not respiration is the main driving force of the systemic venous return in the Fontan circulation

**DOI:** 10.1038/s41598-019-38848-5

**Published:** 2019-02-14

**Authors:** Dominik Daniel Gabbert, Christopher Hart, Michael Jerosch-Herold, Philip Wegner, Mona Salehi Ravesh, Inga Voges, Ines Kristo, Abdullah A. L. Bulushi, Jens Scheewe, Arash Kheradvar, Hans-Heiner Kramer, Carsten Rickers

**Affiliations:** 1 Department of Congenital Heart Disease and Pediatric Cardiology, DZHK (German Center for Cardiovascular Research), partner site Hamburg/Kiel/Lübeck, University Hospital Schleswig-Holstein, Arnold-Heller-Str. 3, Kiel, Germany; 2 Department of Radiology, Brigham and Women’s Hospital and Harvard Medical School, Boston, MA 02115 USA; 3 The Edwards Lifesciences Center for Advanced Cardiovascular Technology, University of California, CA 92697 Irvine, USA; 4 Present Address: Asklepios, Department of General Pediatrics and Adolescent Medicine, St. Augustin, Germany; 5 Present Address: University Heart Center, Adult with Congenital Heart Disease Unit, University Hospital Hamburg-Eppendorf, Martinistrasse 52, 20246 Hamburg Germany

**Keywords:** Congenital heart defects, Magnetic resonance imaging

## Abstract

The Fontan procedure provides relief from cyanosis in patients with univentricular hearts. A major clinical unmet need is to understand whether the venous flow patterns of the Fontan circulation lead to the development of congestive hepatopathy and other life-threatening complications. Currently, there is no consensus on whether heart beat or respiration is the main driving force of venous return and which one affects the periodic flow changes for the most (i. e., pulsatility). The present study, for the first time, quantified respiratory and cardiac components of the venous flow in the inferior vena cava (IVC) of 14 Fontan patients and 11 normal controls using a novel approach (“physio-matrix”). We found that in contrast to the normal controls, respiration in Fontan patients had a significant effect on venous flow pulsatility, and the ratio of respiration-dependent to the cardiac-dependent pulsatility was positively associated with the retrograde flow. Nevertheless, the main driving force of *net* IVC flow was the heart beat and not respiration. The separate analysis of the effects of respiration and heart beat provides new insights into the abnormal venous return patterns that may be responsible for adverse effects on liver and bowel of the patients with Fontan circulation.

## Introduction

Establishing the Fontan circulation has been a major milestone in the history of treatment of congenital heart disease (CHD)^1^. The Fontan procedure separates the pulmonary and systemic circuits and provides relief from cyanosis in patients with univentricular hearts. The Fontan circulation is nowadays routinely established through total cavopulmonary connection (TCPC) procedure^2^ in which blood from the superior and inferior vena cava (SVC, IVC) directly drains into the pulmonary arteries, without the pumping action of a subpulmonary ventricle. The factors affecting systemic venous flow return are heartbeat, respiration, skeletal-muscle pumping function, and patient posture^3,4^. In patients with Fontan circulation, the need for passive pulmonary perfusion leads to an altered systemic venous flow pattern that contributes to an increase in hepatic afterload and splanchnic venous hypertension, which may finally lead to long-term complications, such as failure of Fontan circulation and eventually death^5–10^. Experimental placement of a unidirectional valve within the IVC has been explored to prevent retrograde flow and lower hepatic venous pressure^11^. However, more clinical data is needed to establish this procedure to remedy the long-term complications.

Previous MRI studies to quantify the contribution of heartbeat or respiration on systemic venous flow return suffered from several limitations, such as achieving insufficient temporal resolution of the respiratory cycle, averaging over the cardiac cycle, as well as overlooking the cardiac-respiratory interaction^12–15^. Conventional MRI examinations rely on electrocardiography (ECG) gating using the R-wave as the acquisition trigger. Thus, the effects from respiration are averaged in the acquired data. Direct measurements of the blood flow curve with simultaneous cardiac and respiratory gating has not yet been supported by the commercially-available MRI software packages, which limits our ability to compare the cardiac and respiratory effects on the periodic flow changes (i. e., pulsatility). Currently, there is no consensus whether the pulsatility of the systemic venous return flow due to heart beat or respiration predominates in patients with Fontan circulation. Although several echocardiographic and MRI studies were designed to determine respiratory influence^3,12–15^, none has yet analyzed the simultaneous effect of respiration and heartbeat on pulsatility with adequate temporal resolution.

It has been shown previously that the fluid-dynamic power loss of single-ventricle circulation strongly depends on the pulsatility of venous flow waveforms^16^. Two earlier studies have reported conflicting results whether the pulsatility of the systemic venous return flow in the Fontan circulation is predominantly determined by the heartbeat or respiration^14,15^. To address this question, the present study delineates a novel approach that allows simultaneous analysis of cardiac and respiratory contribution to systemic venous flow return and pulsatility in Fontan patients and normal controls.

## Methods

PC-MRI data from patients with Fontan circulation and normal controls were acquired and analyzed according to a protocol approved by the local ethical committee (file reference A168/07).

### Study groups

A total of 14 patients with different types of univentricular heart were studied 1–14 years after TCPC (intraatrial [n = 13] and extracardiac tunnel [n = 1]) with an age range of 3.1 to 16.6 years: hypoplastic left heart syndrome (HLHS; n = 9), tricuspid atresia (n = 2), double-inlet-left-ventricle (n = 2), criss-cross heart (n = 1). The patients were enrolled for CMR studies as part of their routine post-operative follow up. At the time of CMR, 10 patients had an open fenestration. Sedation for the MRI acquisition was required in 12 patients. No mechanical ventilation was used. All patients were in NYHA class 1 and no patient showed symptoms of a Fontan-associated liver disease of evidence of a ‘failing Fontan situation’ by any means at the time of CMR acquisition. The patients had a median ejection fraction of 55% (range 42–70%), trivial or mild atrioventricular valve regurgitation RGF < = 15% (median 2%, range 0–15%), and a cardiac index of 3.7 (range 2.6–5.5). The normal controls (n = 11) included 5 pediatric (age range 4.2–6.7 years) and 6 adult subjects (age range 24.6–36.1 years). Control subjects were healthy volunteers. Informed consent to this prospective study was provided by all subjects or by their legal guardians. Demographics of all studied subjects are listed in Table 1.

**Table 1:** Overview of demographics (age, body surface area (BSA), heart rate, respiratory rate) and results (amplitudes, pulsatility indices, net and retrograde volumes).

	Fontan circulation (N = 14)	Normal circulation (N = 11)	p-value
**Demographics**			
Age [y]	9.2 ± 4.5	18.4 ± 12.3	0.23
BSA [m^2^]	1.1 ± 0.4	1.4 ± 0.6	0.12
Heart rate [min^−1^]	80.1 ± 11.0	78.9 ± 15.1	0.85
Respiratory rate [min^−1^]	21.8 ± 4.3	17.9 ± 5.7	0.11
**Amplitudes [ml/s]**			
Cardiac	20.7 ± 8.2	79.3 ± 45.8	<0.01
Respiratory	52.1 ± 24.4	26.4 ± 12.4	<0.01
Interaction	11.8 ± 9.2	36.1 ± 13.3	<0.01
**Pulsatility indices**			
Cardiac	0.96 ± 0.37	2.26 ± 0.74	<0.01
Respiratory	2.68 ± 1.56	0.82 ± 0.41	<0.01
**Amplitude Ratios**			
Respiratory-to-cardiac	2.83 ± 1.58	0.40 ± 0.18	<0. 001
**Blood volumes and fractions**			
Net volume [ml/m^2^]	21.4 ± 8.4	28.0 ± 9.8	0.02
*Retrograde volumes [ml*/*m*^2^]			
Physio-matrix	1.4 ± 1.6	1.7 ± 1.53	0.63
ECG gating	0 ± 0	1.23 ± 1.39	<0.001
Respiratory gating	1.3 ± 1.5	0 ± 0	<0.01
*Retrograde vol*. *fractions [%]*			
Physio-matrix	−10 ± 10	−5 ± 4	0.70
ECG gating	0 ± 0	−4 ± 4	<0.001
Respiratory gating	−11 ± 9	0 ± 0	<0.01

### MR Image Acquisition

Cardiac MRI studies were performed on a 3.0 Tesla MR system (Achieva 3.0 T, TX-series, Philips Healthcare, Best, Netherlands) with a 32-channel coil for cardiac imaging. An echo planar imaging (EPI) 2D phase contrast (PC-MRI) sequence was applied for real-time flow measurements. The acquisitions were performed with a field of view of 25 cm × 25 cm, a voxel size of 1.42 mm × 1.42 mm, and a slice thickness of 1 cm. A flip angle α = 20^◦^, and a multi-element receiver coil sensitivity encoding (SENSE) factor of 2 for parallel imaging acceleration were used for EPI imaging. ECG and respiration were sampled with lead electrodes and a respiratory belt. Even though the respiration belt does not measure tidal volume, it provides information on respiratory timing.

**Figure 1:**
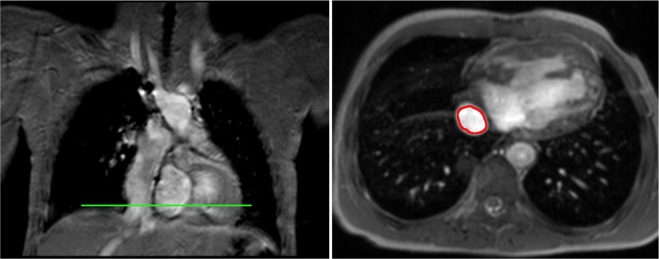
Real-time phase-contrast slice. Left: Coronal view showing the position of the real-time phase-contrast slice in axial orientation (green). Right: Image of the axial slice with the IVC contour (red).

All the data were recorded by the scanner’s physiological monitoring unit (PMU) with a sampling interval of 2 ms. The systemic venous flow was measured at the level of the IVC’s suprahepatic portion with the imaging slices in axial orientation to the vessel, see Fig. 1 (left).

### Flow quantification

Blood flow was quantified from the measured through-plane velocity maps by integration over the IVC vessel area, see Fig. 1 (right). For this purpose, the IVC vessel contours were drawn using a commercially available software (Extended MR WorkSpace, Philips Medical Systems, Best, Netherlands) for each dynamic acquisition. A semi-automatic contour detection was combined with manual contour correction to speed up the process.

### Physio-matrix Approach

Quantifying the physiological effects of heartbeat and respiration on blood flow was achieved by building a two-dimensional data matrix or ‘physio-matrix’, which is a 2D array of flow measurements indexed by the position in the respiratory and cardiac cycles. The physio-matrix determines the variation of blood flow in the course of both, respiration and cardiac cycles. Both cycles were divided into equidistant temporal phases with the R-wave’s peak defining the onset of the cardiac cycle, and the transition between inspiration and expiration labeling the onset of the respiratory cycle.

The physio-matrix was determined by post-processing the real-time data. An analysis program was developed in the scripting-language-based Physics Analysis Workstation (PAW, CERN, Switzerland) to match the PC-MRI flow data with the PMU data, and to compute the physio-matrix.

The respiratory cycle was divided into 40 temporal phases, whereas the cardiac cycle was divided into 10 temporal phases. The temporal resolution of 2D EPI technique led to at least 44 phases per respiratory cycle and 13 phases per cardiac cycle for all the studied subjects. The resolution of the physio-matrix was set in a way not to exceed the resolution of the actual acquisition. The number of 10 phases per cardiac cycle turned out to be sufficient for resolving the triphasic flow pattern. The real-time acquisition was run with a temporal resolution of 48 ms per dynamic scan, and a total acquisition time of 6–7 minutes for 8000 acquisitions. A relatively long total acquisition time was chosen to increase the probability that each combination of cardiac and respiratory phases occurs at least once. The required total acquisition time was estimated from statistical prediction.

The onset of a scan was marked in the physiological data record to allow a synchronization of each flow measurement with the ECG and respiratory curves from the scanner’s PMU. The R-wave peak was automatically recorded by the PMU. The transition from inspiration to expiration was detected off-line by determining the cycle maxima of the respiration belt curve. Local maxima of the respiration curve were detected with a sliding time window of 800 ms, a time interval large enough to extend beyond any plateaus at the peak, and smaller than any observed respiratory cycle length. Accordingly, the respiration curves from the PMU could be subdivided into cycles that began with expiration and ended with inspiration, respectively. Finally, each blood flow data was divided based on the cardiac and respiratory phase, and if more than one flow measurement corresponds to a particular cardiac and respiratory phase index pair in the physio-matrix, that data were averaged.

### Net and retrograde flow

Net blood flow volumes were determined by integrating the physio-matrix over cardiac and respiratory phases, respectively. The net flow volume *V*_*net*_ per cardiac cycle was calculated as:

1 $${V}_{net}=\frac{{\sum }_{i,j}{P}_{i,j}\cdot \Delta {t}_{i}\cdot \Delta {t}_{j}}{\Delta {t}_{i}\cdot {n}_{i}}=\frac{{\sum }_{i,j}{P}_{i,j}\cdot \Delta {t}_{j}}{{n}_{i}}$$

where *i* and *j* are the indices for respiratory and cardiac phase, respectively, and *P*_*i*,*j*_ is a flow-velocity value in the physio-matrix. *∆t*_*i*_ and *∆t*_*j*_ are the respiratory and cardiac phase intervals, respectively, and *n*_*i*_ is the number of respiratory phases. The numerator of the first equation comprises a product of two temporal scales, the cardiac and the respiratory scale. By dividing the numerator by the length of the respiratory cycle Δ*t*_*i*_ *· n*_*i*_, flow volumes were associated to the length of a cardiac cycle, as typically is done for calculation of the stroke volume.

Retrograde flow in the IVC was denoted by negative flow values in the physio-matrix. The retrograde flow volume was calculated by exclusively summing up the physio-matrix’s negative values in the Eq. 1.

In order to simulate the effects of respiratory and cardiac gating on the measurement of retrograde flow, the sum over all fields of the physio-matrix was separated into the sums over respiratory and cardiac phases, respectively. For ECG gating, the sum can be re-written as: *Σ*_*i*,*j*_ *P*_*i*,*j*_ = *Σ*_*j*_ *P*_*j*_^*c*^, where *P*_*j*_^*c*^ = *Σ*_*i*_ *P*_*i*,*j*_ depends only on the cardiac phase index. For respiratory gating, the sum can be re-written as: *Σ*_*i*,*j*_ *P*_*i*,*j*_ = *Σ*_*i*_ *P*_*j*_^*r*^, where *P*_*i*_^*r*^ = *Σ*_*j*_ *P*_*i*,*j*_ depends only on the respiratory phase index. The retrograde flow volume was calculated by summing up the exclusively negative values of *P*_*j*_^*c*^ and *P*_*j*_^*r*^. The net flow volume was independent from the order of the sum, and accordingly was independent from the gating method.

### Generalized Additive Model

The physio-matrix was developed to analyze and compare the flow patterns between the normal and Fontan circulation, and to decompose the flow curves into the components derived from respiration and heartbeat. Such a decomposition into two orthogonal, independent components that exclusively depend on either respiration or heat beat is not necessarily adequate since the interplay between the respiration and heartbeat is physiologically important. Therefore, a generalized additive model (GAM) with periodic, non-parametric smoothers and a functional ANOVA-type decomposition of the structure in form of:

2 $${P}_{i,j}={R}_{i}+{E}_{j}+{I}_{i,j}+Q$$

was used to fit the physio-matrix data, where *R*_*i*_ and *E*_*j*_ are the main components exclusively depending on respiration and cardiac phase, respectively, each with a net area under the curve of zero. *I*_*i*,*j*_ is a tensor product interaction term (in the form of products of the marginal smooths) from which the main components have been excluded, and depends on both respiratory and cardiac phase. *Q* is a constant offset, corresponding to the net flow through the Fontan tunnel. If respiration and heart beat had only independent effects on systemic venous flow, the interaction component, *I*_*i*,*j*,_ would be zero. By scrolling through the positions in the respiratory cycle, the corresponding cardiac curve would be uniformly shifted up and down. By definition, an interaction occurs when the effects of respiration change the cardiac flow waveforms beyond a simple shift.

The GAM analysis allows characterizing the relative flow amplitudes from *R*_*i*_ and *E*_*j*_ originating from the heartbeat and respiration, respectively. Here, the flow amplitude is defined as the difference between the maximal and minimal flow values. The respiratory and cardiac pulsatility indices are defined as the ratio of the respective flow amplitude, and the constant flow term, *Q*. The distinct partition of the net flow, *Q*, corresponding to the constant term in the GAM model into the respiratory and cardiac contributions requires additional information. Absolute values of the flow contributed by the heartbeat and respiration can be estimated by using the respiratory resting state as the reference (to be described in the following section). The respiratory-to-cardiac amplitude ratio was determined as the ratio of the respiratory amplitude divided by the IVC flow’s cardiac amplitude. The respiratory-to-cardiac flow amplitude ratio is equal to the ratio of the respiratory and cardiac pulsatility index. The GAM fits were performed with the statistical computing software, R^17^, whose underlying theory has been well-described before^18^.

During the respiratory resting state, the intrathoracic pressure equals atmospheric pressure with no pressure gradient opposing the flow in the SVC and IVC. The flow value at the corresponding flow plateau averaged over all cardiac phases may serve as an estimate of the cardiac net contribution to IVC blood flow. The respiratory contribution to IVC blood flow was determined from the difference between the total net flow, *Q*, and the purely cardiac contribution to net blood flow. To define the flow waveform’s plateau corresponding to the respiratory resting position, we selected the portion where the local slope from an 8-point linear fit was less than 5% of the mean slope between the local maxima and minima of the flow waveform.

### Statistical Methods

Statistical analyses were performed with the computing software, R^17^. Given that the null hypothesis assumed that each component of the generalized additive model (GAM) is zero, the significance of each flow component was determined using the Wald test^19^. The difference between the respiratory-to-cardiac blood flow amplitude ratios for normal and Fontan circulation was analyzed with the Mann-Whitney-U-Test. Spearman’s rho was used to analyze the association of the respiratory-to-cardiac-amplitude flow ratio with retrograde flow fraction.

### Ethics approval and consent to participate

This study has been approved by the local ethical committee (file reference A168/07, Ethik-Kommission, Arnold-Heller-Straße 3, Haus 9, Kiel, Germany, 2014). Methods were carried out in accordance with the relevant guidelines and regulations. Informed consent to participate was obtained from the participants or their legal guardians.

## Results

**Figure 2:**
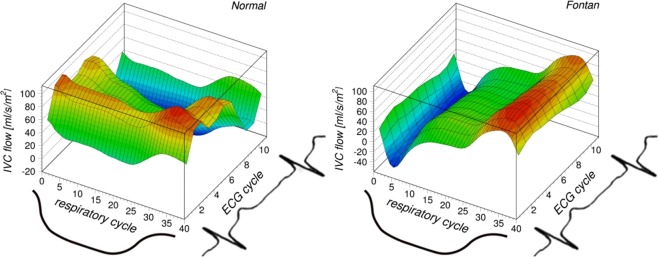
Comparison of the physio-matrix in a healthy control subject (left) and a patient with Fontan circulation (right). The diagrams show how IVC flow depends on the respiratory and cardiac cycle. The color scheme emphasizes the changes of flow values: high flow values are indicated in red, whereas low values are indicated in blue.

A triphasic venous flow pattern of the normal controls corresponding to the atrial filling (first peak), ventricular filling (second peak) and atrial contraction (downward deflection) is shown in the left panel of Fig. 2. In contrast, the right panel in Fig. 2 shows along the respiratory axis a respiratory flow pattern of patients with Fontan circulation with downward deflection at the beginning of expiration, a plateau during respiratory resting period, followed by a peak during inspiration.

### Blood flow amplitudes and pulsatility

**Figure 3:**
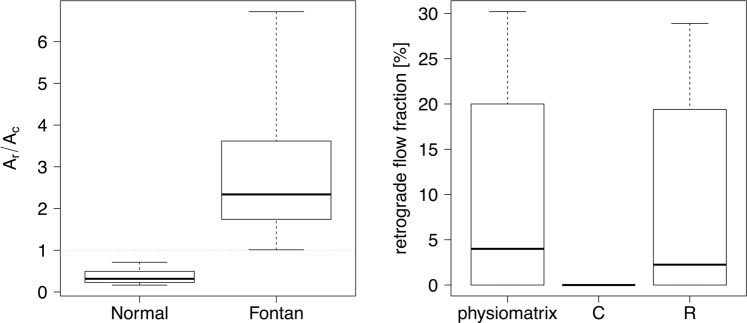
Respiratory-to-cardiac amplitude ratio and retrograde flow. Left: Ratio of respiratory (A_r_) and cardiac (A_c_) systemic venous flow amplitudes was significantly different in normal controls (n = 11) versus Fontan patients (n = 14). Right: Retrograde flow fraction for patients in Fontan circulation as determined with three methods: simultaneous gating using the physio-matrix, ECG gating (C), and respiratory gating (R).

The components of periodic flow changes related to respiration and heartbeat (GAM fit analysis) were both found to be statistically significant (p < 0.05) in all the studied subjects. The significance was determined for each patient and normal control, based on the null hypothesis that each component of the GAM is zero. In patients with Fontan circulation, flow amplitudes due to respiration were significantly higher compared to the flow amplitudes related to the heartbeat (Wilcoxon sign rank test, p < 0.001). In normal controls, the flow curve pattern showed a predominantly cardiac-dependent variation compared to the respiration dependent variation (Wilcoxon sign rank test, p < 0.001). The resultant respiratory-to-cardiac amplitude ratio was higher than 1:1 for Fontan patients, but lower for the control subjects. The difference between the respiratory-to-cardiac amplitude ratios in Fontan patients and normal controls were highly significant according to a Mann-Whitney-U-test (p < 0.01). The range of data showed no overlap between the patients (2.83:1 ± 1.58:1, range 1.01:1–6.72:1) and normal controls (0.4:1 ± 0.18:1, range 0.16:1–0.71:1). Pediatric and adult normal controls were not further sub-divided into separate subgroups – pediatric and adult subjects in the control group did not have any significantly different respiratory-to-cardiac amplitude flow ratios according to a Mann-Whitney-U-test (p = 0.33, pediatric controls: 0.41:1 ± 0.21:1 range 0.21:1–0.71:1; adult controls: 0.38:1 ± 0.18:1 range 0.16:1–0.67:1) despite the high median age difference of 25 years. These findings are shown in Fig. 3 (left). Correlations of the respiratory-to-cardiac amplitude ratio of Fontan patients with valvular ejection fraction, atrioventricular valve regurgitation or cardiac index were not significant (p > 0.05).

### Retrograde flow and net flow

In Fontan patients, the retrograde fraction of venous flow estimated with the physio-matrix averaged 10.0% ± 11.2%, with a range from 0.0% to 30.2%. Exclusive respiratory gating led to a slight underestimation of retrograde flow fraction from 0% to 3%. ECG gating led to the measurement of a zero-net retrograde flow in all patients. In normal controls, the physio-matrix provided an estimate of retrograde fraction of venous flow that ranged from 0.0% to 10.4%, with a mean ± SD of 5.4% ± 4.3%. Exclusive ECG gating in normal controls led to a slight underestimation ranging from 0% to 5%. With exclusive respiratory gating, no retrograde flow was detected in control subjects. These findings are shown in Fig. 3 (right).

Correlations of the fractional retrograde IVC flow of Fontan patients with valvular ejection fraction, atrioventricular valve regurgitation or cardiac index were not significant (p > 0.05).

**Figure 4:**
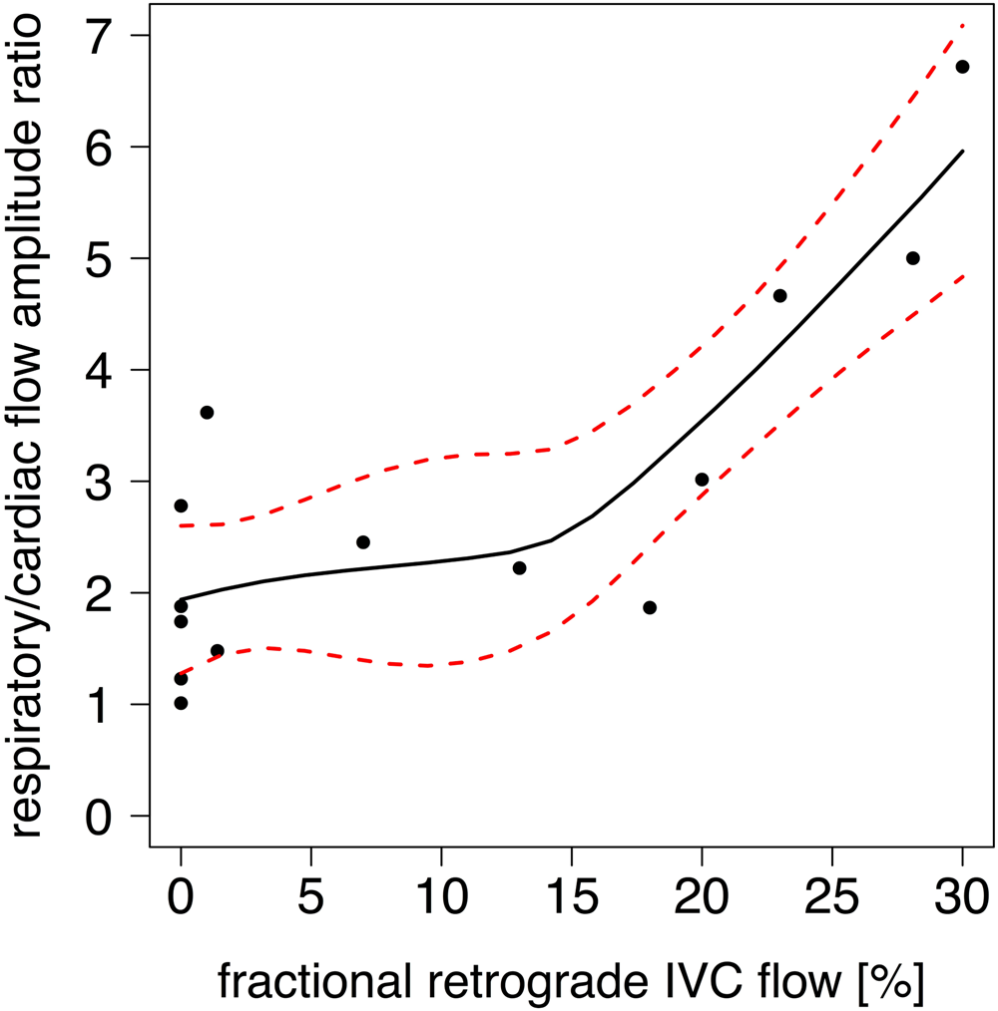
Correlation of the respiratory-to-cardiac-amplitude ratio with the fractional retrograde IVC flow (rho = 0.79, p < 0.01). Data is overlaid with a spline fit and a 95% confidence band.

In Fontan patients, the respiratory-to-cardiac amplitude ratio was found to be associated with the retrograde flow fraction, as illustrated in Fig. 4. Of note is that for retrograde flow fractions greater than ~15%, the respiratory-to-cardiac amplitude ratio rose relatively rapidly, compared to a relatively constant mean of the ratio for retrograde flow fractions below ~15%. (A retrograde flow fraction of 15% corresponds approximately to the 97.5 percentile level for retrograde flow fraction in control subjects.).

Six of the 14 patients showed a flow plateau during the respiratory resting phase. In these patients, respiration had an average effect of 6% (ranges between 0% and 15%) on the net systemic venous flow.

## Discussion

Quantification of the effects of heartbeat on systemic venous flow based on exclusive ECG gating results in averaging flow over the respiratory cycle, and similarly, exclusive respiratory gating leads to averaging over the cardiac cycle^15^. Our method, which simultaneously measures both cardiac- and respiration-dependent flow components, avoids averaging out periodic flow changes due to one of these components. Such an averaging method may result in an incorrect assessment of venous hemodynamics in the Fontan circulation. Using this novel approach, the ratio of the simultaneously measured pulsatility due to respiration and heartbeat in the systemic venous system can be analyzed with sufficient temporal resolution. This method provides new insights into the abnormal venous return patterns potentially responsible for adverse effects on liver and bowel of patients with Fontan circulation.

### Physio-Matrix Approach

The physio-matrix developed here allows a comprehensive assessment of venous flow hemodynamics. This approach quantifies the ratio of respiration-dependent and cardiac-dependent pulsatility, and retrograde and net flows. The 3D surface plots of the physio-matrix allow both qualitative and quantitative comparisons between the normal and Fontan circulation, as shown in Fig. 2. In Fontan patients, the most prominent effects on periodic flow changes depend on respiration.

### Pulsatility

**Figure 5:**
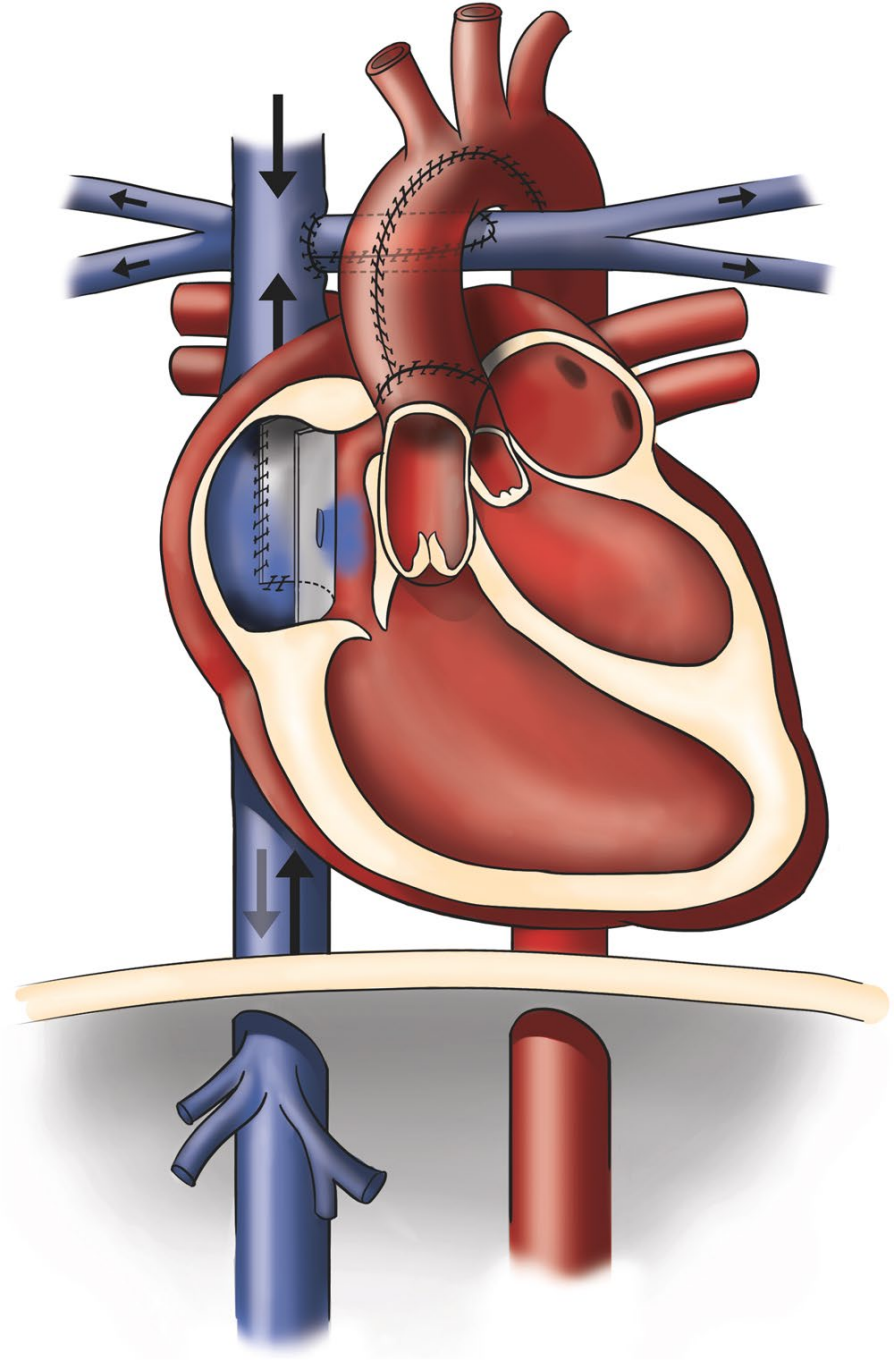
Schematic drawing of the Fontan circulation as established in a patient with hypoplastic left heart syndrome. The right ventricle (RV) serves as the systemic ventricle that pumps blood through the aorta. The drawing shows the Fontan circulation in an anatomy where the IVC is guided through the right atrium by an intraatrial tunnel with fenestration. Systemic venous return from inferior (IVC) and superior (SVC) vena cava drains directly into the right (RPA) and left (LPA) pulmonary arteries without interconnection of a cardiac pump.

**Figure 6:**
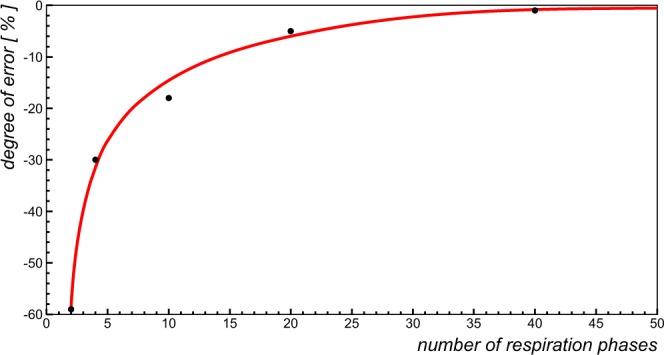
Illustration of the systematic error in measuring respiratory blood flow amplitudes when the respiratory cycle is divided into different numbers of respiratory phases. The determination was performed for a patient with Fontan circulation during 50 respiratory phases as repeated with gradually reduced numbers of phases. The error is indexed to a number of 50 phases. Using 40 phases underestimates respiratory amplitudes by ~one percent whereas the use of two phases introduces an error of ~60%.

The abnormal systemic venous flow patterns in the Fontan circulation can be explained by the passively perfused lungs, see Fig. 5. As reflected by the significantly higher respiratory-to-cardiac blood flow amplitude ratio, cardiac effect on systemic venous flow pulsatility was found lower and respiratory influence on pulsatility was found higher than in the control subjects. Although previous studies investigated the respiratory pulsatility of the systemic venous flow in the Fontan circulation using MRI^12–15^ or echocardiography^3^, those analyses were limited by low temporal resolution, as only two^3,12,15^ to four phases^13^ per respiratory cycle were used. Despite the high temporal resolution of echocardiography, the cited study^3^ evaluated only two temporal phases by integration over inspiration and expiration time-intervals. Figure 2 shows that the narrow flow peaks with large slopes require much higher temporal resolution. In our study, we divided the respiratory cycle into 40 phases. Based on our analysis, any less than 20 temporal phases are likely insufficient for assessing the effect of respiration on the flow curves (Fig. 6). While a temporal resolution of 40 respiratory phases results in a systematic error for the respiratory blood flow pulsality amplitude of about 1%, this error dramatically increases with lower temporal resolution, as illustrated in Fig. 6.

In this study, the respiratory-to-cardiac blood flow amplitude ratio was used for the first time to explore the simultaneous respiratory and cardiac effects on the systemic venous return flow pulsatility. The respiratory-to-cardiac amplitude ratio predicts the degree of retrograde flow, an indicator of energy loss, as shown in Fig. 4. Retrograde flow in the IVC results in elevated portal vein pressures as demonstrated with an *in vitro* Fontan mock circulatory system by implanting a bovine valved conduit^11,20^. Elevated hepatic pressures can lead to morbidity and mortality after Fontan operation. Hepatic autoregulation of parenchyma perfusion cannot compensate adequately if portal vein pressures exceed 20 to 25 mm Hg, leading to reduced hepatic perfusion (impaired arterial buffer function) and hepatic injury^21^.

### Retrograde flow

The only echocardiographic study that investigated the retrograde flow in the Fontan circulation found retrograde-to-antegrade flow ratios of 0.06 ± 0.11 for the subhepatic IVC flow and 0.27 ± 0.17 for the hepatic venous flow^3^. Previous MRI studies have not reported the retrograde flow in the Fontan circulation, presumably because exclusive cardiac gating cannot detect retrograde flow, as shown in Fig. 3 (right) and Table 1. Our results show that the determination of retrograde flow using gated MRI techniques requires respiratory gating in Fontan patients, whereas conventional ECG gating can be sufficient in healthy control subjects (see Table 1). In Fontan patients, the retrograde flow fraction in the suprahepatic portion of the IVC averaged 10 ± 10% and the distribution of values shows a long tail reaching up to 30% of net flow. Retrograde flow in Fontan patients can only be detected by respiratory gating. Therefore, it is safe to conclude that retrograde flow in Fontan patients is mainly driven by respiration. In contrast, the retrograde flow fraction in normal controls does not exceed 10%, and is exclusively driven by the heartbeat. This strong association of retrograde flow with respiration in the Fontan circulation has not been studied with similar detail before.

### Net systemic venous return

Presently, respiratory maneuvers such as breath-holding, Valsalva maneuver, and Müller maneuver have been used to determine the contribution of respiration to the net systemic venous return^22–24^. With the exception of the breath-holding technique in a recent MR-study^14^, none of the other respiratory maneuvers has been tried to define the contribution of respiration to net venous return in the Fontan circulation. The novelty of our approach is that it allows determining the contribution of respiration to net flow under free-breathing conditions by comparing the overall net flow with the average flow during the respiratory resting position. The use of breath-hold techniques does only allow measurement of mean IVC flow in the Fontan circulation but cannot assess respiration-dependent pulsatility and retrograde flow towards the liver.

### Respiration or heartbeat?

The question of whether respiration or heartbeat provides the dominant effect on the systemic venous flow in the Fontan circulation can be considered from two perspectives: with respect to blood flow amplitudes and flow pulsatility, or in terms of net blood flow. The venous flow amplitudes in the Fontan circulation significantly change during the respiratory cycle, while the heartbeat plays a minor role – a reversed pattern compared to the normal circulation. Considering the *net* flow, the respiratory contribution can be neglected in Fontan patients. Despite the interposed lung, the heart is still the driving force of the net flow in Fontan circulation. This finding is in agreement with the previous findings of the net flow between free-breathing and breath-hold acquisitions of an adult cohort^14^. Our analysis shows that respiration leads to a significant increase in flow amplitudes and can even results in a retrograde flow pattern, which may impose a long-term burden to the abdominal organs, e. g. the liver.

Respiration therapy with deeper inspiration has been recommended for Fontan patients. It aims to increase intrathoracic pressure amplitudes to improve lung perfusion^25^. Since respiration drives retrograde flow in the Fontan circulation, such a therapy may have a detrimental hemodynamic effect by increasing hepatic afterload.

### Limitations

The group of normal controls was rather small (n = 11). Heart and respiratory rates of normal controls and Fontan patients were not significantly different (p = 0.85 and p = 0.11, respectively). Although the differences in heart rate and respiration rate did not appear to be statistically-significant, we cannot exclude type II errors. Furthermore, a 1.2 bpm difference in heart rate, even if statistically significant, is unlikely to be physiologically relevant. To exclude a type II error for a heart rate effect size of 1.2 bpm (mean SD of 13 bpm) with a probability of 90%, a couple of thousand patients per group is required, which is unrealistic for this rare congenital malformation. Regarding the respiratory rate, an effect size on the order of 4 breaths/min (SD = 15) could have been significant with a group size of 40~ or larger. Indeed, a higher respiratory rate in Fontan patients compared to normal controls, could reflect the dependence of the lung perfusion in the Fontan circulation on intrathoracic pressure changes and the respiration rate. Nevertheless, the relative difference in the respiratory rates is still relatively small, compared to the difference between the respiratory-to-cardiac amplitude ratios of control subjects and Fontan patients. The statistically significant difference between the respiratory-to-cardiac amplitude ratios in normal controls and Fontan patients was not associated with the small and presumably insignificant differences in heart rate and respiratory rate of both groups.

In patients in which flow reached no plateau during the respiratory resting phase, respiratory contributions to net flow could not be determined. In normal controls, respiratory flow curves were relatively flat. Thus, the respiratory resting state could not be located in the curves and cardiac contributions to net flow were not determined in normal controls.

Since we only performed flow analysis in the suprahepatic IVC, we may not extend the results to the SVC flow. Finally, the physio-matrix approach in its current form is time-consuming as it had to segment approximately 8000 images. However, recent advances using machine learning and in particular deep learning for cardiac MR image segmentation^26,27^ will help overcome this limitation in the near future.

## Conclusion

Respiration is not a dominant driving force of the net systemic venous flow in Fontan patients, but has a major influence on pulsatility and retrograde flow. Their respiratory flow amplitudes are significantly higher than in normal controls, and the respiratory-to-cardiac flow amplitude ratio predicts a higher degree of retrograde flow, a marker of increased hepatic afterload. Future studies will have to evaluate if the increased respiratory-to-cardiac amplitude ratios are related to anatomical variants of the TCPC^28^ and whether it can be used to predict adverse sequelae of the Fontan circulation.

## Data Availability

The data from this study are available from the authors on reasonable request.
