# Publisher Correction: Heart beat but not respiration is the main driving force of the systemic venous return in the Fontan circulation

**DOI:** 10.1038/s41598-019-50842-5

**Published:** 2019-10-01

**Authors:** Dominik Daniel Gabbert, Christopher Hart, Michael Jerosch-Herold, Philip Wegner, Mona Salehi Ravesh, Inga Voges, Ines Kristo, Abdullah A. L. Bulushi, Jens Scheewe, Arash Kheradvar, Hans-Heiner Kramer, Carsten Rickers

**Affiliations:** 1 Department of Congenital Heart Disease and Pediatric Cardiology, DZHK (German Center for Cardiovascular Research), partner site Hamburg/Kiel/Lübeck, University Hospital Schleswig-Holstein, Arnold-Heller-Str. 3, Kiel, Germany; 2 Department of Radiology, Brigham and Women’s Hospital and Harvard Medical School, Boston, MA 02115 USA; 3 The Edwards Lifesciences Center for Advanced Cardiovascular Technology, University of California, CA 92697 Irvine, USA; 4 Present Address: Asklepios, Department of General Pediatrics and Adolescent Medicine, , St. Augustin, Germany; 5 Present Address: University Heart Center, Adult with Congenital Heart Disease Unit, University Hospital Hamburg-Eppendorf, Martinistrasse 52, 20246 Hamburg, Germany

Correction to: *Scientific Reports* 10.1038/s41598-019-38848-5, published online 14 February 2019

In the original version of this Article, the author Mona Salehi Ravesh was incorrectly indexed. This error has now been corrected.

